# Supplementary material for: The prevalence and topography of spinal cord demyelination in multiple sclerosis: a retrospective study
Source: Acta Neuropathol. 2024 Mar 9;147(1):51. doi: 10.1007/s00401-024-02700-6 (PMC10924711; doi:10.1007/s00401-024-02700-6)
Supplement: Supplementary file 1 — Supplementary file1 (DOCX 16 KB) [file 401_2024_2700_MOESM1_ESM.docx]

Supplementary Table 1. Antibodies and Conditions Used for Immunohistochemistry

| Antibody | Clone | Classification | Antigen Retrieval | Dilution | Block | Incubation | Supplier | Catalogue |
| --- | --- | --- | --- | --- | --- | --- | --- | --- |
| PLP | plpc | Mouse Monoclonal | Microwave, Citrate pH 6 | 1:1000 (TBST) | None | 1hr RT | BioRad | MCA839G |
| CD68 | PGM1 | Mouse Monoclonal | Microwave, Citrate pH 6 | 1:100 (TBST) | None | 1hr RT | DAKO | M0876 |

PLP=proteolipid protein, TBST=tris-buffered saline, 0.05% triton-X, RT=room temperature
