# Supplementary figures and images for: The prevalence and topography of spinal cord demyelination in multiple sclerosis: a retrospective study
Source: Acta Neuropathol. 2024 Mar 9;147(1):51. doi: 10.1007/s00401-024-02700-6 (PMC10924711; doi:10.1007/s00401-024-02700-6)

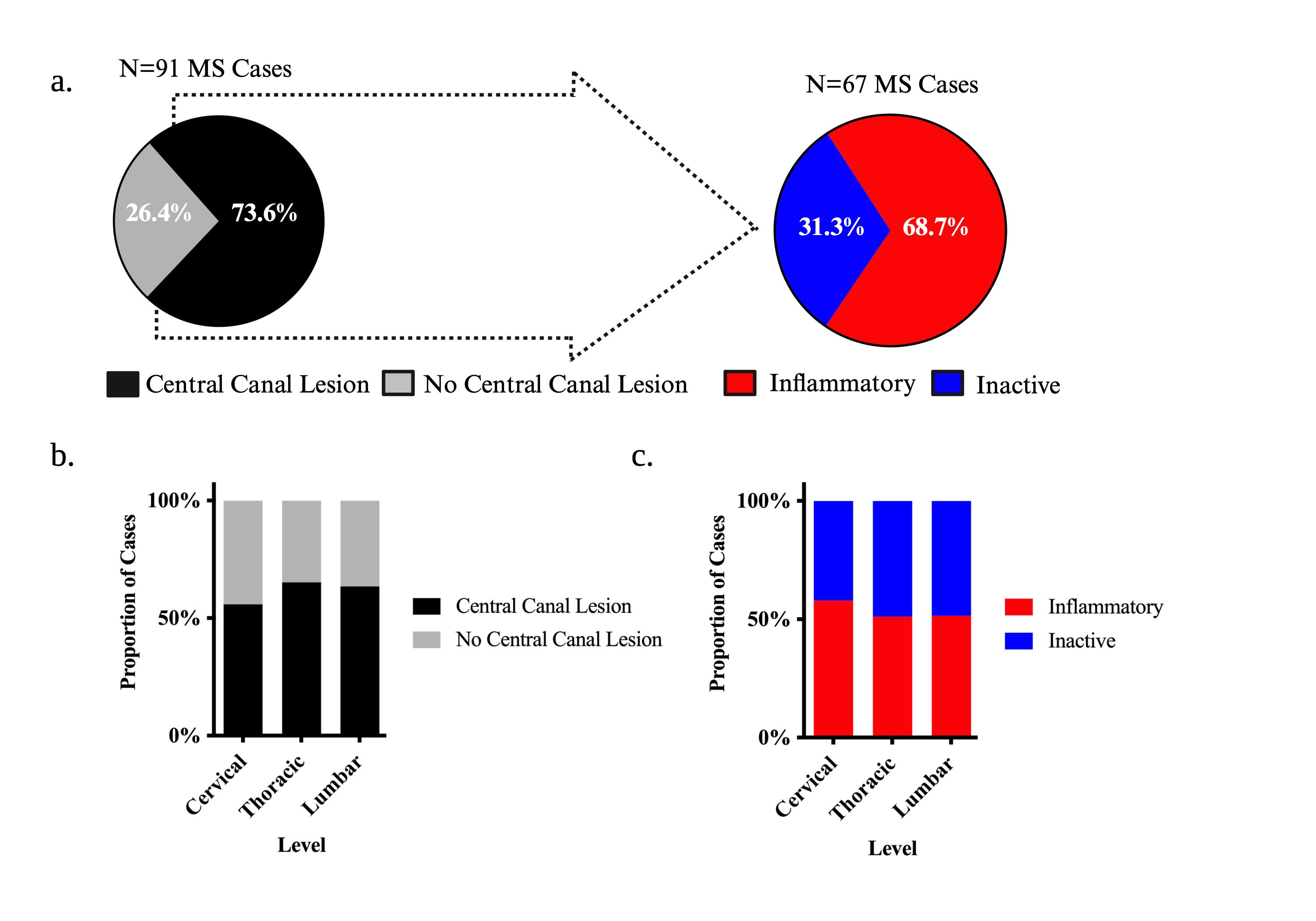

Supplement: Supplementary file 3 — Supplementary file3 (TIFF 402 KB) Supplementary Fig. 2. Prevalence of Demyelination in the Central Canal of the MS Spinal Cord. (a) Pie charts highlighting the observed proportion of cases with central canal lesions and those that harboured at least 1 inflammatory (active or mixed/active inactive) central canal lesion. (b-c) Stacked bar charts depicting the proportion of MS cases that harboured central canal lesions at each level of the spinal cord irrespective of stage (b) and classified by the presence of inflammation (c). Proportions represent observed values, and the asterisks indicate significant post-hoc pairwise comparisons following logistic mixed modelling and multivariate adjustment for multiple comparisons (*p<0.05; **p<0.01; ***p<0.001; ****p<0.0001) [file 401_2024_2700_MOESM3_ESM.tiff]
